# Prevention of Oxidative Stress-Induced Retinal Pigment Epithelial Cell Death by the PPAR*γ* Agonists, 15-Deoxy-Delta 12, 14-Prostaglandin J_2_

**DOI:** 10.1155/2008/720163

**Published:** 2008-03-16

**Authors:** Jason Y. Chang, Puran S. Bora, Nalini S. Bora

**Affiliations:** ^1^Department of Neurobiology and Developmental Sciences, University of Arkansas for Medical Sciences, Little Rock, AR 72205, USA; ^2^Department of Ophthalmology, University of Arkansas for Medical Sciences, Little Rock, AR 72205, USA; ^3^Department of Microbiology and Immunology, University of Arkansas for Medical Sciences, Little Rock, AR 72205, USA

## Abstract

Cellular oxidative stress plays an important role in retinal pigment epithelial (RPE) cell death during aging and the development of age-related macular degeneration. Early reports indicate that during phagocytosis of rod outer segments, there is an increase of RPE oxidative stress and an upregulation of PPAR*γ* mRNA in these cells. These studies suggest that activation of PPAR*γ* may modulate cellular oxidative stress. This paper presents a brief review of recent studies that investigate RPE oxidative stress under various experimental conditions. This is followed by a detailed review on those reports that examine the protective effect of the natural PPAR*γ* ligand, 15d-PGJ_2_, against RPE oxidative stress. This agent can upregulate glutathione and prevent oxidant-induced intracellular reactive oxygen species accumulation, mitochondrial depolarization, and apoptosis. The cytoprotective effect of this agent, however, is not shared by other PPAR*γ* agonists. Nonetheless, this property of 15d-PGJ_2_ may be useful in future development of pharmacological tools against retinal diseases caused by oxidative stress.

## 1. AGE-RELATED MACULAR DEGENERATION: POSSIBLE INVOLVEMENT OF RPE

Age-related macular degeneration (AMD) is the leading cause of legal blindness in individuals 50 years of age or older in the United States and developed countries. AMD can be divided into two major forms as follows: (i) nonneovascular form, also known as “*dry*” or “*nonexudative*” form; as clinical findings of this form include drusen and abnormalities of the retinal pigment epithelium (RPE) and (ii) neovascular form, also known as “*wet*” or “*exudative*” form, which is defined by the appearance of choroidal neovascularization with subsequent subretinal fibrosis or disciform scarring. Patients with drusen larger than 63 *μ*m in diameter (termed “*soft drusen*”) have a high risk of developing choroidal neovascularization [1].

There is evidence that pathological alterations of RPE around macula area may be partially responsible for the development of AMD [2, 3]. Clinical abnormalities of RPE in AMD include clumping and atrophy of these cells. RPE is involved in the ingestion of photoreceptor outer segments and the general health of photoreceptors. As a result, pathological changes of RPE can lead to photoreceptor cell death and visual impairment. Study with human cadaver eyes indicates that there is an age-dependent RPE apoptosis as evidenced by TUNEL staining [4]. A separate study further indicates that eye specimens from patients with AMD show statistically more macular RPE apoptosis than those without AMD [5].

## 2. POSSIBLE ROLES OF OXIDATIVE STRESS IN AMD

Retina is exposed to a combination of sunlight, high concentrations of polyunsaturated fatty acids, and high oxygen environment. It is proposed that reactive oxygen species (such as hydrogen peroxide, superoxide anion, hydroxyl radicals, and singlet oxygen) are constantly generated in this environment. As a result, oxidative stress is believed to have an important role in RPE apoptosis and in the development of AMD [2, 3].

An increase of oxidative stress in RPE is associated with an increase of cellular catalase, metallothionein [6], and glutathione S-transferase [7], which should serve as a protective mechanism to decrease the cytotoxicity caused by H_2_O_2_ and other reactive oxygen species. This protective mechanism declines with age. For example, a study analyzing metallothionein levels in RPE of macular region showed a significant (68%) decrease in *aged* donors (mean age = 80-year-old) as compared to those from *younger* donors (mean age = 58-year-old) [8]. A separate report also concluded that there was an age-dependent decrease of catalase activity in RPE [9]. These studies suggest that RPE cells in the elderly are more susceptible to oxidative stress-induced damage.

## 3. STUDIES OF OXIDATIVE STRESS ON RPE: PREVENTION BY PHARMACOLOGICAL AGENTS

Given the observations that RPE might be the prime targets for oxidative stress, a number of studies are conducted to study this issue. A majority of research use direct oxidative agents, such as hydrogen peroxide (H_2_O_2_) or t-butylhydroperoxide (tBH), to initiate cellular oxidative stress, as further discussed below. Other conditions of experimental oxidative stress include: intense light [10–12], iron [13], and oxidative metabolites that are toxic to cells, such as A2E [14, 15], acrolein [16], and oxysterols [17–19].

By using H_2_O_2_ or tBH as the direct source of oxidative stress on RPE, a number of studies focus on strategies to build up cellular defense mechanisms against the insult. Several reports explore the importance of cellular antioxidative enzymes, such as catalase [20], glutathione-S-transferase [21, 22], superoxide dismutase [23], and methionine sulfoxide reductase [24]. Growth factors including lens epithelium-derived growth factor [25], keratinocyte growth factor [26], and pigment epithelium-derived factor [27] are also protective against oxidative stress. Other proteins that can enhance RPE antioxidative mechanism against H_2_O_2_ include bcl-2 [28], alpha B-crystallin [29], melatonin [30], and poly(ADP-ribose) polymerase [31].

In addition to those protein factors discussed above, many investigators seek the use of small-molecule pharmacological agents to prevent RPE damage caused by H_2_O_2_ or tBH. Examples of these pharmacological agents include: (R)-alpha-lipoic acid [32], 17-beta-estradiol [33], flavonoids [34], and L-carnitine [35]. The endogenous PPAR*γ* ligand, 15-deoxy-delta-12,14-prostaglandin J_2_(15d-PGJ_2_), is also very effective in preventing RPE oxidative stress, as further discussed below.

## 4. PREVENTION OF OXIDATIVE STRESS-INDUCED RPE DEATH BY 15D-PGJ_2_

15d-PGJ_2_, a prostaglandin derivative, is normally present in tissues at low levels (<1 nM), but can reach high concentrations during infection and inflammation [36]. Under in vitro conditions, it can be induced by chemical [37] or physical [38] stress. It has a very potent anti-inflammatory effect [39]. For example, it is a potent inhibitor of macrophage [40–42] and microglia [43–45] activation.

During RPE ingestion of rod outer segments, there is a generation of H_2_O_2_ [6, 46] and a 10-fold upregulation of PPAR*γ* mRNA [47]. Based on these observations, it is likely that PPAR*γ* is involved in RPE cellular responses toward H_2_O_2_. One can hypothesize that PPAR*γ* agonists should modulate cellular defense against oxidative stress.

**Figure 1:**
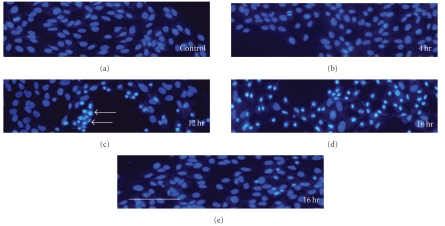
*Prevention of H_2_O_2_-induced nuclear condensation by 15d-PGJ_2_.* The human RPE cell line ARPE-19 cells were treated with 1.5 mM H_2_O_2_ for various periods of time, and then processed for nuclear staining by bisbenzimide (Hoechst 33258) to identify apoptotic cells [48]; (a): untreated cells; (b): 4 hours; (c): 12 hours; (d): 16 hours after treatment. Arrows in (c) point to representative cells with condensed nuclei, an indication of apoptosis. (e): Cells were pretreated with 1 *μ*M 15d-PGJ_2_ overnight, followed by 1.5 mM H_2_O_2_ for 16 hours (without 15d-PGJ_2_). The number of apoptotic cells was greatly reduced by 15d-PGJ_2_. Scale bar: 100 *μ*m.

We reported earlier that the PPAR*γ* agonist,15d-PGJ_2_, protected H_2_O_2_-induced RPE cell death [48]. With primary human RPE cells, pretreatment of cells overnight with 15d-PGJ_2_ dose-dependently prevented H_2_O_2_-induced cytotoxicity, such that the viability raised from *∼*25% (H_2_O_2_ only) to *∼*80% of control. Maximal protection was observed at *∼*2 *μ*M 15d-PGJ_2_. Similar protection was made in the human ARPE-19 cell line. While H_2_O_2_ caused significant nuclear condensation, a sign of apoptosis; this was largely prevented by 1 *μ*M 15d-PGJ_2_ (see Figure 1). However, it should be mentioned that the protective effect by 15d-PGJ_2_ was not shared by other PPAR*γ* agonists, such as ciglitazone, azelaoyl PAF, or LY171883. These results raised the possibility that the protective effect by 15d-PGJ_2_ was not mediated through PPAR*γ* activation. This idea was supported by other investigators, as further discussed below.

The cytoprotective effect of 15d-PGJ_2_ on H_2_O_2_-treated RPE was further studied by Qin et al. [49]. These investigators confirmed that 1 *μ*M 15d-PGJ_2_ effectively prevented H_2_O_2_-induced cell death. Other PPAR*γ* agonists, such as AGN195037 or Roziglitazone, had no protective effects. Importantly, reduction of PPAR*γ* by siRNA did not block the protective effect of 15d-PGJ_2_. This set of experiments together with those described above strongly suggests that 15d-PGJ_2_ protect RPE cells through a PPAR*γ*-independent mechanism. Some properties of 15d-PGJ_2_ are independent of PPAR*γ* activation, as reviewed by Straus and Glass [39].

Subsequent studies by Qin et al. [49] indicated that 15d-PGJ_2_ could upregulate glutamylcyteine synthetase, the rate-limiting enzyme that regulates glutathione (GSH) synthesis. These investigators reported that 15d-PGJ_2_ at 1-2 *μ*M induced GSH levels to *∼*300% of control. With 1 *μ*M 15d-PGJ_2_, the maximal induction occurred at 18–24 hours after treatment. This GSH induction appeared to depend on JNK and p38 pathways because inhibitors of these pathways greatly reduced GSH induction by 15d-PGJ_2_. Induction of GSH by 15d-PGJ_2_ is also observed in other cell types [37, 50, 51]. Since intracellular GSH is very important in cellular defense against oxidative stress, the induction of GSH should have an important role in the protective effect caused by 15d-PGJ_2_ treatment. Even though induction of heme oxygenase-1 (HO-1) was associated with cytoprotective effects of 15d-PGJ_2_ in other studies [52], this enzyme had no roles in the protection observed in this experimental system.

If 15d-PGJ_2_ greatly induced intracellular GSH, one would expect that this agent should reduce oxidant-induced intracellular reactive oxygen species generation. Indeed, we reported earlier that 15d-PGJ_2_ could reduce H_2_O_2_- and tBH-induced reactive oxygen species in human ARPE-19 cells [53]. For example, pretreatment of cells with 1 *μ*M 15d-PGJ_2_ reduced 1 mM H_2_O_2_-generated reactive oxygen species to *∼*80% of untreated cells challenged with H_2_O_2_. Similar reduction was observed in cells challenged with tBH. This reduction apparently was enough to keep free radical levels under a critical threshold, thus rendering cells survive an otherwise detrimental oxidant insult.

**Figure 2:**
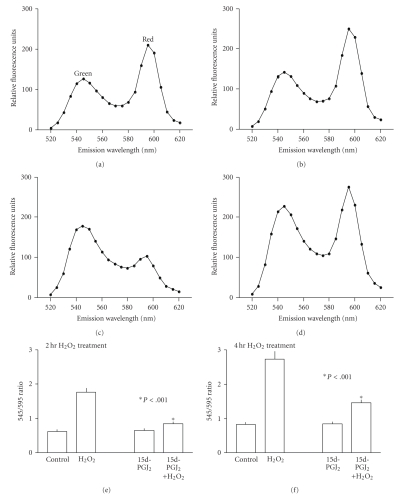
*Prevention of H_2_O_2_-induced mitochondrial membrane depolarization by 15d-PGJ_2_.* Binding of the JC-1 dyes to mitochondria leads to the appearance of two peaks. The green peak (at *∼*545 nm) represents JC-1 monomers of this dye. The red peak (at *∼*595 nm) represents JC-1 aggregates, which is caused by the negative charge of mitochondrial membrane. Depolarization of mitochondrial membrane causes a shift in the emission spectrum from red to green color, which can be quantified by a fluorescence plate reader. The relative intensity of these two peaks is a measurement of relative mitochondrial potential such that a higher ratio represents more mitochondrial membrane depolarization. (a)–(d): The JC-1 emission spectra between 520 nm to 620 nm were determined for cells under various conditions [53]; (a): untreated cells; (B): cells treated with 1 *μ*M 15d-PGJ_2_ overnight; (c): cells treated with 1.5 mM H_2_O_2_ for 2 hours; (d): Cells treated with 1 *μ*M 15d-PGJ_2_ overnight, then with 1.5 mM H_2_O_2_ (without 15d-PGJ_2_) for 2 hours. Note H_2_O_2_ caused a shift of the relative intensity of the peaks, and 15d-PGJ_2_ pretreatment restored membrane potential to a condition closer to untreated cells. (e)-(f): Cells were pretreated with 1 *μ*M 15d-PGJ_2_ overnight, then with 1.5 mM H_2_O_2_ (without 15d-PGJ_2_) for 2 hours (e) or 4 hours (f); then the 545/595 emission intensity ratios were determined. Note in either 2-hour or 4-hour treatment, H_2_O_2_ caused an increase of the 545/595 emission intensity ratio, indicating mitochondrial depolarization. 15d-PGJ_2_ pretreatment restored the ratio to that similar to control value (*P* < .001 between H_2_O_2_-treated and 15d-PGJ_2_+H_2_O_2_-treated cells in (e) and (f)).

Our study further indicated that 15d-PGJ_2_ helped RPE cells to maintain mitochondrial integrity [53]. This is significant because mitochondria are intimately involved in apoptosis. Oxidative stress can induce mitochondria dysfunction, which is a critical event that leads to cytochrome c release and subsequent activation of caspases, a group of enzymes that executes apoptosis [54, 55]. An important event associated with mitochondrial dysfunction is a drop of mitochondrial membrane potential (ΔΨ*m*), that is, mitochondrial depolarization. This event initiated by oxidative stress was largely prevented by 1 *μ*M 15d-PGJ_2_ (see Figure 2). This is likely to prevent cytochrome c release and subsequent activation of the apoptosis pathway.

## 5. CYTOPROTECTIVE VERSUS CYTOTOXIC EFFECTS OF 15D-PGJ_2_

In addition to those studies described above regarding the protective effect of 15d-PGJ_2_ against oxidative stress on RPE, this agent is cytoprotective toward other retinal cells. For example, Aoun et al. [56] reported that glutamate could induce oxidative stress and cell death in the rat retinal ganglion cell line, RGC-5 cells. This cell death was prevented by 1–5 *μ*M 15d-PGJ_2_. Outside of retina, 15d-PGJ_2_ was effective in preventing glutamate-induced cell death of primary cortical neurons [51]. Both groups attributed the protective effect through the antioxidative property of 15d-PGJ_2_. In this respect, it should be noted that this agent can also prevent cell death caused by toxic metabolites of oxidative stress. For example, we reported earlier that 15d-PGJ_2_ prevented cytotoxicity of oxysterols, toxic cholesterol metabolites generated under oxidative stress [57]. The cytoprotective effect of 15d-PGJ_2_ in other experimental systems were also described in reports by Kawamoto et al. [58] and Itoh et al. [59].

It is clear now that 15d-PGJ_2_ can induce intracellular oxidative stress [60, 61]. It is likely that this agent at low concentrations (1–5 *μ*M) can cause low levels of oxidative stress, thus inducing the build up of cellular defense mechanisms against oxidative stress. However, at high concentrations, this agent can cause severe oxidative stress and cell death [60, 61]. Induction of apoptosis by this agent was reported in several cell types [62–64]. This interesting bifunctional property of 15d-PGJ_2_ has been reported [50], and is a subject of review by Na and Surh [65]. This also prompts a recent microarray study analyzing the regulation of prosurvival and prodeath genes by this agent [66].

## 6. CONCLUDING REMARKS

**Figure 3:**
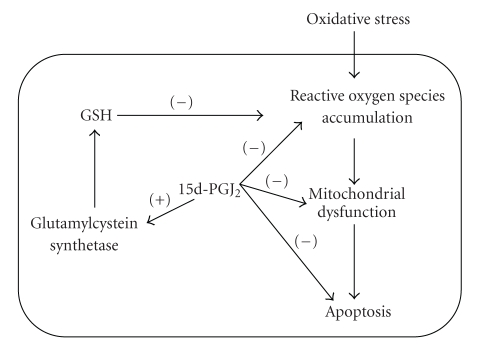
*Protective effects of 15d-PGJ_2_ against oxidative stress.* Oxidative stress on RPE cells can lead to intracellular accumulation of reactive oxygen species. This can result in mitochondrial dysfunction, which in turn causes activation of the apoptosis pathway. Current data suggests that 15d-PGJ_2_ can block each of these events. One mechanism that causes this protection is through upregulation of GSH synthesis by activation of the glutamylcystein synthetase. There is a possibility that other cytoprotective mechanisms are also activated that lead to prevention of apoptosis. This remains to be studied.

Oxidative stress is believed to play an important role in RPE cell death during aging and the development of age-related macular degeneration. During phagocytosis of rod outer segments, there is an upregulation of PPAR*γ* in RPE cells. The natural PPAR*γ* ligand 15d-PGJ_2_ has a potent protective effect for RPE under oxidative stress. This agent can upregulate GSH and prevent oxidant-induced intracellular reactive oxygen species accumulation, mitochondrial depolarization, and apoptosis (see Figure 3). There is also evidence that 15d-PGJ_2_ can prevent glutamate-induced death of cultured retinal ganglion cells. Current data suggests that this cytoprotection is not mediated through the activation of PPAR*γ*. The antioxidative property of 15d-PGJ_2_ may be useful in future development of pharmacological tools against retinal diseases caused by oxidative stress.

Finally, based on anti-inflammatory effects of 15d-PGJ_2_, we would like to speculate that this agent might be effective in the treatment of other ocular diseases such as idiopathic autoimmune anterior uveitis. To confirm our hypothesis, we intend to explore the effect of 15d-PGJ_2_ on experimental autoimmune anterior uveitis (EAAU) which serves as an animal model of idiopathic human autoimmune anterior uveitis [67, 68].
